# Use of test accuracy study design labels in NICE’s diagnostic guidance

**DOI:** 10.1186/s41512-019-0062-9

**Published:** 2019-09-05

**Authors:** M. Olsen, Z. Zhelev, H. Hunt, J. L. Peters, P. Bossuyt, C. Hyde

**Affiliations:** 1 Amsterdam University Medical Centers, Department of Clinical Epidemiology, Biostatistics and Bioinformatics, Amsterdam Public Health Research Institute, Meibergdreef 9, 1105 AZ Amsterdam, The Netherlands; 2 Exeter Test Group, Institute of Health Research, University of Exeter Medical School, St Lukes Campus, Exeter, EX1 2LU UK

**Keywords:** Diagnostic test accuracy study design, Labels, Terminology, NICE diagnostic guidance

## Abstract

**Background:**

A variety of study designs are available to evaluate the accuracy of tests, but the terms used to describe these designs seem to lack clarity and standardization. We investigated if this was the case in the diagnostic guidance of the National Institute of Care and Health Excellence (NICE), an influential source of advice on the value of tests.

**Objectives:**

To describe the range of study design terms and labels used to distinguish study designs in NICE Diagnostic Guidance and the underlying evidence reports.

**Methods:**

We carefully examined all NICE Diagnostic Guidance that has been developed from inception in 2011 until 2018 and the corresponding diagnostic assessment reports that summarized the evidence, focusing on guidance where tests were considered for diagnosis. We abstracted labels used to describe study designs and investigated what labels were used when studies were weighted differently because of their design (in terms of validity of evidence), in relevant sections. We made a descriptive analysis to assess the range of labels and also categorized labels by design features.

**Results:**

From a total of 36 pieces of guidance, 20 (56%) were eligible and 17 (47%) were included in our analysis. We identified 53 unique design labels, of which 19 (36%) were specific to diagnostic test accuracy designs. These referred to a total of 12 study design features. Labels were used in assigning different weights to studies in seven of the reports (41%) but never in the guidance documents.

**Conclusion:**

Our study confirms a lack of clarity and standardization of test accuracy study design terms. There seems to be scope to reduce and harmonize the number of terms and still capture the design features that were deemed influential by those compiling the evidence reports. This should help decision makers in quickly identifying subgroups of included studies that should be weighted differently because their designs are more susceptible to bias.

**Electronic supplementary material:**

The online version of this article (10.1186/s41512-019-0062-9) contains supplementary material, which is available to authorized users.

## Introduction

Critical appraisal of studies is an essential element of evidence-based medicine. A first step in an evaluation is to identify the type of studies used to generate the evidence. Depending on the design used, the validity may be threatened. Some study designs are more likely than others to generate evidence that is free from systematic errors, i.e. bias.

The selection, recruitment and flow of participants—hence, the study design—is one of the most important pieces of information needed to assess validity in diagnostic test accuracy (DTA) studies. However, DTA study designs and their intrinsic biases differ from those of, say, drug trials. The validity of a DTA study depends in particular on how and where participants were recruited and, among other features, whether the complete study group or subgroups underwent both the index test and reference standard. In DTA studies, ideally, a single, consecutive group of participants undergoes both the index test and the reference standard. Meta-epidemiological studies have convincingly shown that some types of study design lead to biased estimates of the performance of diagnostic tests [1–3].

A clear description of the crucial design features of DTA studies, preferably with a concise label, may help in characterizing a study. In comparative studies of the effectiveness of interventions, for example, randomized clinical trials offer stronger evidence than studies without randomization, since randomization of a large group of participants reduces bias due to confounding. As such, the ability to distinguish randomized from non-randomized studies is crucial for the evaluation of any study [4]. Likewise, in etiological questions, labels from epidemiology, such as “case-control study” and “cohort study”, provide crisp and clear information of how the identification of study participants was organized [5]. Unfortunately, to our knowledge, there is no set of comparable standardized labels or terms for describing diagnostic accuracy study designs. This absence may generate confusion.

In systematic reviews of DTA studies, the QUADAS-2 instrument is the most often used tool to evaluate the risk of bias and to identify concerns about the applicability of findings of DTA studies [6] and the STARD checklist encourage clear and transparent reporting [7]. However, none of these tools offers standardized terminology for describing the study designs. Moreover, the QUADAS-2 uses the case-control terminology in the item that evaluates patient selection (the risk of spectrum bias). Consequently, any confusion arising from this is not limited to readers of the primary studies. It also has potential implications for the assessment of bias and applicability of studies included in the evidence synthesis that informs clinical guidelines and health policies.

The National Institute for Care and Health Excellence (NICE) is well recognized for developing evidence-based clinical guidelines. Since 2011, the NICE Diagnostic Assessment Programme (DAP) has developed specific diagnostic guidance. DAP was among the first international bodies to develop guidelines and to offer guidance for DTA evidence synthesis. Although the programme follows high international standards for evidence synthesis, the terminology and labels used to describe the evidence varies and includes labels non-specific to DTA (e.g. “prospective cohort or cross-sectional studies, or retrospective case-control studies”) [8]. Hence, without the ability to clearly communicate different threats, there is a risk that guidance committees may miss important aspects and implications of study designs.

We investigated the use of study design labels to describe test accuracy studies in the development of NICE diagnostic guidance. We extracted data to describe the range of study design terms and labels in the NICE evidence reports of diagnostic accuracy reviews and guidance documents and to investigate what labels were used when studies were weighted differently because of their design.

## Methods

### Guidance identification and eligibility criteria

A NICE diagnostic guidance is developed in several steps. In short, an external academic assessment group performs a systematic review of the evidence (i.e. the diagnostic assessment report, DAR), for a given question issued by NICE. The report is evaluated by the NICE Diagnostics Advisory Committee (DAC), who develops the guidance based on the report’s findings alongside any other evidence emerging. These recommendations are finally confirmed and released by NICE in a separate document (i.e. the NICE diagnostic guidance, DG). All NICE guidance and DARs are publicly available at the NICE home page, from which we identified the report/guidance pair that were published from the NICE Diagnostic Assessment Programme.

Guidance was eligible for inclusion in the study if the corresponding DAR included a diagnostic question and a clear diagnostic test accuracy review. Report/guidance pairs were excluded if they had only reviewed evidence for monitoring, predictive and/or prognostic questions, since corresponding studies have designs, features and labels that differ from those of diagnostic accuracy studies.

### Data extraction

A data extraction form was developed by one reviewer and piloted independently by two other reviewers on three reports/guidance documents, which were subsequently excluded from the final analysis. From the remaining 17 report/guidance pairs, five reviewers (ZZ, HH, JP, CH, MO) independently extracted data from pre-specified sections in which we expected labels would be used.

To identify the range of labels used in the reports, we extracted data from the following DAR sections: abstract, scientific summary, the sections reporting the eligibility criteria and quality assessment process, and the discussion and conclusion section. From the guidance document, we extracted data from the following sections: recommendation, evidence and committee discussion. For the second objective (what labels were used when studies were weighted differentially), we extracted data from the DAR sections: scientific summary, eligibility criteria, discussion and conclusion. In the guidance, we scrutinized sections on evidence, committee discussion and recommendations for future research. A label or term was extracted if it referred to any study design characteristic or feature (specific investigative methods or strategies used in a study).

### Analysis

We listed all labels, including what proportion of DAR and guidance documents used labels in each scrutinized section, and made a descriptive analysis for the range of labels. Label interpretations were discussed between authors and then assigned to categories, based on their level of specificity in describing DTA studies.

For our second objective, we discussed and included any instance in which a design feature was ranked or considered to have a higher (or lower) methodological preference or status, for the given report or guidance. For example, in DG17, the use of “within-study comparisons” was preferred over other comparisons made across different groups (Additional file 1: Table S5).

## Results

**Fig. 1:**
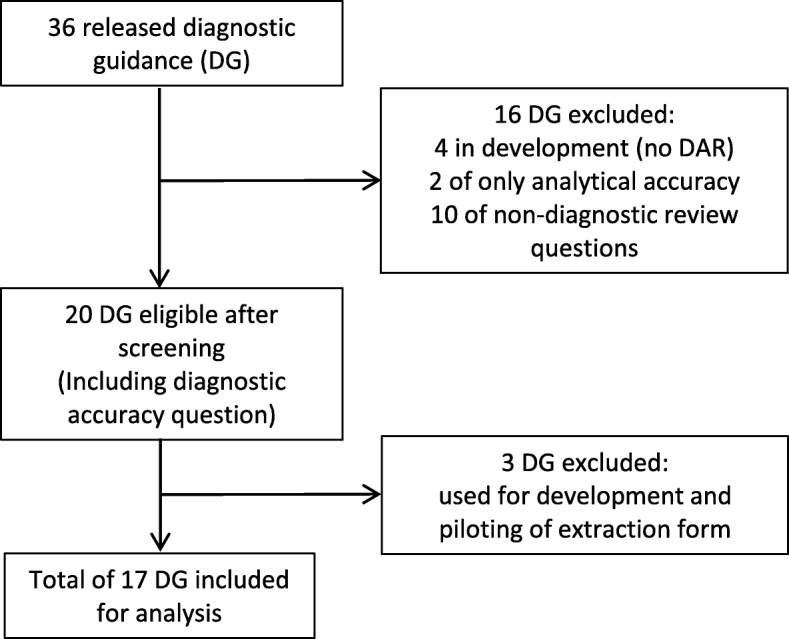
Flow diagram of the diagnostic guidance

On 6 July 2018, we retrieved all guidelines published as part of the NICE DAP since its inception. The programme had at that time released a total of 36 diagnostic reports with guidance, of which 20 (56%) were found eligible. After exclusion of the three reports used for development and piloting of the data extraction form, we were able to include a total of 17 (47%) guidelines and their corresponding DARs (Fig. 1). The guidelines covered a variety of topics, ranging from immunochemical tests in colorectal cancer to devices for measuring exhaled nitric oxide in asthma and assessment of CT scanners for cardiac imaging (Additional file 1: Table S1).

### Range of labels and study design features

In the 17 report/guidance pairs, we found a total of 53 unique labels (Additional file 1: Table S2). We categorized them as follows, based on their relevance for DTA studies (Table 1).

**Table 1:** Labels grouped by DTA-specificity

Label	Interpretation	Similar/synonym labels
1. DTA-specific labels (*n* = 19)		
1.1 DTA-informative (*n* = 6)		
Comparative performance	Comparison of minimum two index test/strategies [3].	Comparative (diagnostic) accuracy studies
*Direct comparisons*^1^	Comparison of two index test/strategies within the same group/population (i.e. with the same set of eligibility criteria).	Head to head comparisons; within-study comparisons
(Prospective cohort) randomized to either the index test or comparator^2^	Randomization to either the index test or reference standard/comparator.	Randomized head-to-head comparisons
(Prospective cohort) receiving the comparator and at least one index test with follow up^3^	All participants receive both an index test and the reference standard/comparator and are followed up for a final outcome measure/status.	
Reference standard positive studies	Only participants with a positive reference standard test receive the index test.	
*Single/two-gate diagnostic studies*	Including one (single set of eligibility criteria) or two/multiple groups (separate sets of eligibility criteria) for comparison [9].	Two-study cohorts
1.2 DTA-descriptors (*n* = 9):		
Single/*multi-centre study*	Participants are sampled from a single or from multiple centres.	Large, multi-centre prospective (UK) study; two-centre
Primary care study	Participants are sampled from settings in the primary (vs. secondary or tertiary) care.	
Consecutive recruitment	Participants are sampled consecutively (vs. random or convenient sampling) [2].	
Symptomatic study	Including only symptomatic participants.	
(*Single-gate studies*) *recruiting populations at high risk*	Including only high risk participants.	
Population (-based) studies	Including/sampling participants that represent the target/study population [9].	(Multi-centre) community-based study
*Pro- or retrospective studies*	Data collection (or recruitment) was planned before (prospective study) the index test and reference standard were performed or after (retrospective study) (STARD) [7].	Prospective cohort studies; prospective, consecutive cohort study; prospective, international multicentre
Retrospective analysis (of prospective database)	Post-hoc or non-pre-specified statistical analysis.	
Discordant case analysis^4^	A study that excludes discordant cases in the analysis.	
1.3 General DTA labels (*n* = 4)		
(*Diagnostic*) *cohort studies*	A diagnostic cohort study in which “individuals are enrolled before the final outcome (presence or absence of the target condition) is known” [9] (i.e. a single cohort/group of suspected participants).	(Retrospective) cohort studies
(Diagnostic) case-control studies/design	“(…) studies in which the disease status is already known before the index test is performed”. “(…) the reference test is applied only to a subsample of the participants with or without the target condition”. [9]	
(Diagnostic) *cross-sectional studies*	Comparison of the result of the index test with that of the reference standard in the same participant at the same time. [9]	
(Diagnostic) *observational studies*	A DTA-study that only make use of observational data e.g. re-interpretation/review of existing tests results.	
2. Umbrella terms (*n* = 4):		
Diagnostic accuracy studies	A study reporting diagnostic accuracy performance measures.	(Large prospective cohort) studies collecting diagnostic accuracy data
*End-to-end studies*	A diagnostic accuracy study that directly links test results to patient outcomes (such as RCTs) [8].	
*Long-term studies* (following patients for several years)	A study that includes follow-up of any given outcome measure/status.	
Pilot studies	A study that is testing/piloting a setting/feasibility, often with a small sample size.	
3. Non-DTA-specific labels (*n* = 9)		
Test-treat trials	A study in which a test guides the treatment strategy.	
Controlled clinical trials	A clinical trial including a control (comparator) group to the intervention group.	
Cluster-randomized controlled trial	A study that randomizes clusters of participants to either intervention or control group.	
Derivation study	A study that focuses on the development of a prediction model (contra validation of a model).	
Observational studies	A study that only includes observational data (non-intervention data).	
(Observational) cohort studies	A study using data from all cohort-members (in contrast to case-control study that uses data from an outcome-selected subset of the source population) [5, 10].	
Prospective, two cohorts (feasibility, validation)	A study with two separate cohorts recruited prospectively.	
*Randomized controlled trials* (*RCTs*)	A study with randomization of participants to either intervention group(s) or a control (comparator) group (vs. non-randomized).	(Large multi-centre) RCT
Validation study	A study that focuses on validation (of an existing/developed model).	
4. Unclear labels (*n* = 3)		
Diagnostic studies with a control group		
(Multi-centre) tracker study		
Mixed design (of within-study comparisons)		

The table shows the classification of the labels identified and their most likely interpretations. The *labels in italic* are labels (also) used in the guidance

^1^Elsewhere, other possible interpretation could be comparison of index test and reference test within the same patient (i.e. fully paired) [11]

^2^In DTA terminology, the term “comparator” usually refers to one of two index tests (in a comparative study). However, in this case, the “comparator” seems to refer to other tests that are used for the comparison of the index tests (which is used when a commonly accepted/implemented reference standard is absent) [12]. Other possible interpretation could be randomization to one of several index tests or randomization to two different test-sequence strategies

^3^In other contexts of DTA-studies, “follow-up” can imply the clinical follow-up compose the reference standard itself (i.e. the final outcome, diagnosis, is established by follow-up). This is also referred to as a “delayed type cross-sectional study” [13]. Yet, it can also mean that any given reference standard is performed at a later time point (delayed) than the index test

^4^This could also mean studies that identify discordant cases

One group contained labels considered specific for DTA-studies (*n* = 19, 36%). An example is “reference standard positive studies”, in which only participants with a positive reference standard test receive the index test. A second group consisted of umbrella labels, as these described more global study types but did not provide specific information of DTA designs (*n* = 4, 8%). An example would be “long-term studies”. A third group were non-specific labels, meaning that they were judged as more likely referring to other research types (*n* = 9, 17%). Examples here are “test-treat trials”, in which management is guided by test results. A fourth group contained unclear labels: labels for which it was difficult to decide how they should be interpreted within the context of DTA studies (*n* = 3, 6%). An example was a “(multi-centre) tracker study” (Table 1).

Focusing only on the DTA-specific labels, we could classify these further into subgroups based on the type of information they provided. One subgroup was considered DTA-informative, as these refer to the overall flow of participants in the study (*n* = 6, 32%). The distinction between “single-gate and two-gate studies” falls in this category. A second subgroup was formed by labels that were referring to more specific design features. These could be considered additional DTA-descriptors that do not refer to an overall design (*n* = 9, 47%). An example is a “single centre study” or a “symptomatic study”, in which only symptomatic participants are included. A third subgroup of DTA-specific labels contains labels that are very general, typically derived from other study types, often from etiologic epidemiology studies (*n* = 4, 21%). An example is “diagnostic cohort studies”.

Only a few labels could be referenced to existing literature and we had to rely on assumptions for the interpretation of most of the labels (Table 1).

We also explored what study design features were described. We identified 4 main design domains and 12 design features (including descriptors) among the 19 DTA-specific labels (Additional file 1: Table S3). From the DTA-specific labels, we also identified a set of labels that lead to confusion when describing DTA studies. In Additional file 1: Table S4, we list all terms that, in our opinion, should preferably be avoided as other more specific terms exist.

### Variations in label interpretations

We observed discrepancies in the use and interpretation of some DTA labels, both among and within reports. The “single-gate” and “two-gate” labels, for example, were introduced by Rutjes and colleagues to describe the use of a single or two sets of eligibility criteria. They did so as an alternative for the confusing use of “cases” and “controls” in DTA studies. In the NICE reports, however, “single-gate” and “two-gate” labels could refer to disease status (known or unknown), to the study flow (if all patients or subgroups receive both tests) or to a combination of these (Table 2).

**Table 2:** Single- and two-gate interpretations

Reference	Text	Variations in interpretation
Rutjes et al. [9]	“(…). All patients pass through a single-gate: a single set of criteria for study admission, typically defined by the clinical presentation.” “(…) We refer to this as a “two-gate design using healthy controls”. Two different sets of inclusion criteria (gates) are used: one for the diseased and another for the non-diseased participants.”	A single- and two-gate is defined by the number of eligibility criteria (sets).
DAR8	“The majority of included studies comply with a single-gate design, a single sample of individuals with unknown metastatic status was assessed by both the diagnostic test under scrutiny and the reference standard.”	A single-gate study is defined by a single sample with (unknown) disease status and all participants receiving both reference standard and index test.
DAR20	“Single-gate: A study design in which only patients with the target condition are recruited.” “(…), single-gate studies, that is, studies in which only patients with the target condition (suspected sepsis) were recruited.”	A single-gate study is defined by inclusion of only patients by (known) disease status. Later, participants with the target condition are described as suspected individuals.
DAR27	“Single-gate study: Where a single sample of individuals is assessed by both the index test and reference standard.” “Two-gate study: Studies which employ separate sampling schemes for diseased and non-diseased participants, with both groups being assessed by the index test.”	A single-gate is defined by both (a single set of) eligibility criteria and study flow (receiving both tests). Two-gate study is defined by (two sets of) eligibility criteria, (known) disease status and study flow both groups receiving the index test.
DG33	“Single-gate study: Study design where participants’ disease status is unknown and the index test result is evaluated against the reference standard to confirm the diagnosis”	A single-gate is defined by inclusion of participants by only (unknown) disease status and receiving both tests.

The table shows variations in the interpretations of the single- and two-gate labels from included DG. The first row shows the citations from the original definition made by Rutjes et al. [9]

### Frequency of study design labels (between sections)

**Fig. 2:**
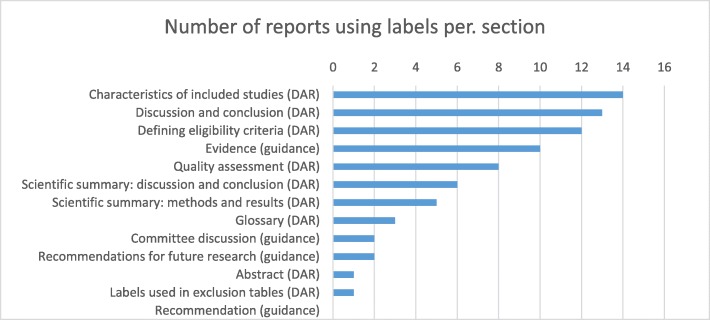
Frequency of labels used in DARs and guidance

Overall, labels were most often used in the methods and results section of the DARs, including the description of the characteristics of included studies, discussion and conclusion, and in reporting eligibility criteria. Less frequent was the use of labels in the DAR’s exclusion tables, quality assessment and summary sections (incl. scientific summaries, abstract and glossary). Except for the evidence section, labels were seldom used in the final guidance of recommendations, committee discussion and recommendations for future research (Fig. 2).

### Labels used to distinguish studies by designs

We also explored if labels had been used to differentiate studies by design features. This would be the case if a report had given a different weight to different study design features and had used labels to indicate this distinction.

From the DARs, we found a total of 7 (41%) reports that had used labels when differentiating between study design features. The terminology varied from being clear, with description such as “Single-gate diagnostic studies with random or consecutively recruited participants were considered the optimal design” and “The preferred data for this review are derived from within-study comparisons of intervention and comparator test pathways”, to less clearly terminology, such as “The majority of studies which have adjusted for TAB (tissue allocation bias), have taken a conservative approach by excluding affected samples, which we consider to be a reasonable practice” (Additional file 1: Table S5). We found no guidelines in which the committees had used labels to emphasize differences between studies.

## Discussion

Study design labels can help in the clear and transparent communication of the way a study was organized. However, the lack of an agreed upon terminology for DTA studies acts as a barrier for effective communication. This lack of clarity may lead to confusion, both in primary and secondary research.

We investigated the use of study design labels in the evidence reports (DARs) and final guidance in the NICE Diagnostic Assessment Programme and were able to confirm that labels and terms are used very inconsistently. We found that, from a total of 53 unique labels, approximately one third were specific to DTA-studies. Yet, these ranged from labels that were informative of the essential DTA design features, over labels that we classified as descriptors, to labels that were general, mostly adopted from other study types, and with an ambiguous meaning within the context of DTA study designs.

Our study has several limitations and uncertainties. We did not perform additional verification of the data extraction. However, we believe that a label is a relatively easily identifiable item to extract and the risk of having missed labels is low. It was not always clear if the labels belonged to the overall clinical effectiveness review or specifically to the DTA review. Some of the labels we extracted may not exclusively be intended to describe the DTA studies. We also relied on a rather broad definition of study design labels, which may be sensitive to individual interpretation. As we focused on the variation of labels and not on the frequencies of each label, we do not consider this to have affected the results.

Among the DTA-informative labels, we found ambiguity, as it was not always clear for us how to interpret the various labels. In particular, two (related) terms were unclear. The term “comparator” has previously been defined as referring to a second or third index test in a comparative accuracy study, in which all index tests are evaluated against a single reference standard [3, 11]. However, in the scenario where there is no agreed upon clinical reference standard, this imperfect reference standard is sometimes also referred to as “comparator”. A second source of confusion was the use of the term “randomization”. This lack of clarity in the distinction between the index test(s), comparator and reference standard makes it difficult to understand what arms participants had been allocated to.

We also observed variation in the use of “single-gate” and “two-gate” designs, although this label is unique to DTA studies. Some reports defined the terms by inclusion of a single or multiple groups/sets of eligibility, while in others they were defined by disease status (known or unknown) and/or who received both tests.

The second group of descriptors referred to features that are easier to interpret, such as “single centre” or “multicentre studies”. One set of terms was ambiguous: the use of “prospective” and “retrospective” as descriptors of studies. These could refer to one of several different components: recruitment, data collection and analysis. In some cases, it was clear from the context what authors were referring to, as in “prospective cohort randomized to either the index test or comparator”, “retrospective analysis”. In other cases, the interpretation was less clear.

To our knowledge, no other form of DTA guidance provides more standardized labels of study designs. Neither the US AHRQ Methods Guide for Medical Tests Reviews or the Cochrane Handbook for DTA Reviews provides standardized terms [8, 14]. Hence, the lack of consistent and standardized DTA-terminology may play a key role in this use of ambiguous labels. For example, terms that have been used in epidemiology to describe the timing of recruitment and data collection are not always straightforward when applied in DTA studies. This includes terms as case-control study, longitudinal study or historical cohort study. One difficulty lies in the fact that DTA studies are essentially cross-sectional. The use of “borrowed” labels makes any interpretation of the exact DTA design difficult. For this reason, we believe that the use of such terms as “retro-”and “prospective”, “longitudinal”, “cohort” and “case-control” should be avoided and that more specific alternatives are needed for describing DTA designs.

Only 7 reports had used labels when they differentiated between studies, while no guidance document had. This shows that the use of labels in communicating important differences is limited, which consequently makes interpretation difficult. Committees are often assembled from experts who will read the reports in detail. Yet, without standardized design terminology for clear communication in general, the attention to different degrees of validity in DTA studies in the overall assessment may be hindered.

## Conclusion

Taken together, our results show that there is a lack of standardized terms and labels to characterize differences in DTA-study design. This presents a barrier to informative reporting of primary studies and hinders attempts at making valid synthesis of the available evidence which, in turn, may have consequences for developing evidence-based guidelines and other recommendations for policy makers. Although attempts to standardize language are notoriously difficult, the research community should consider investing more efforts in developing and consistently using descriptive labels, to avoid confusion and misinterpretation.

## Additional file

Additional file 1: **Table S1.** List of included guidance. **Table S2.** List of unique labels identified. **Table S3.** Identified key design domains and features. Legend: The table shows all design domains and features (left column) that were identified from the DTA-specific labels (right column). **Table S4.** List of terms to avoid and alternative preferred terminology. Legend: The table shows the design features (column one) for which we found confusing terms (column two). **Table S5.** Examples of when labels were used to differentiate studies. (DOCX 28 kb)

## Data Availability

The datasets used and analyzed during the current study are available from the corresponding author on reasonable request.

## Abbreviations

DAC: Diagnostics Advisory Committee
DAP: Diagnostic Assessment Programme
DAR: Diagnostic assessment report
DG: Diagnostic guidance
DTA: Diagnostic test accuracy
NICE: National Institute of Care and Health Excellence
