# Supplementary material for: Use of test accuracy study design labels in NICE’s diagnostic guidance
Source: Diagn Progn Res. 2019 Sep 5;3:17. doi: 10.1186/s41512-019-0062-9 (PMC6727490; doi:10.1186/s41512-019-0062-9)
Supplement: Supplementary file 1 — Table S1. List of included guidance. Table S2. List of unique labels identified. Table S3. Identified key design domains and features. Legend: The table shows all design domains and features (left column) that were identified from the DTA-specific labels (right column). Table S4. List of terms to avoid and alternative preferred terminology. Legend: The table shows the design features (column one) for which we found confusing terms (column two). Table S5. Examples of when labels were used to differentiate studies. (DOCX 28 kb) [file 41512_2019_62_MOESM1_ESM.docx]

SUPPLEMENTARY

**Table S1.** List of included guidance

| **Guidance Title** | **DG reference** |
| --- | --- |
| New generation cardiac CT scanners (Aquilion ONE, Brilliance iCT, Discovery CT750 HD and Somatom Definition Flash) for cardiac imaging in people with suspected or known coronary artery disease in whom imaging is difficult with earlier generation CT scanners | **DG3** |
| SonoVue (sulphur hexafluoride microbubbles) – contrast agent for contrast-enhanced ultrasound imaging of the liver | **DG5** |
| SeHCAT (tauroselcholic [75 selenium] acid) for the investigation of diarrhoea due to bile acid malabsorption in people with diarrhoea-predominant irritable bowel syndrome (IBS-D) or Crohn's disease without ileal resection | **DG7** |
| Intraoperative tests (RD‑100i OSNA system and Metasin test) for detecting sentinel lymph node metastases in breast cancer | **DG8** |
| Faecal calprotectin diagnostic tests for inflammatory diseases of the bowel | **DG11** |
| Measuring fractional exhaled nitric oxide concentration in asthma: NIOX MINO, NIOX VERO and NObreath | **DG12** |
| Myocardial infarction (acute): Early rule out using high-sensitivity troponin tests (Elecsys Troponin T high-sensitive, ARCHITECT STAT High Sensitive Troponin-I and AccuTnI+3 assays) | **DG15** |
| Diagnosing prostate cancer: PROGENSA PCA3 assay and Prostate Health Index | **DG17** |
| VivaScope 1500 and 3000 imaging systems for detecting skin cancer lesions | **DG19** |
| Tests for rapidly identifying bloodstream bacteria and fungi (LightCycler SeptiFast Test MGRADE, SepsiTest and IRIDICA BAC BSI assay) | **DG20** |
| PlGF-based testing to help diagnose suspected pre-eclampsia (Triage PlGF test, Elecsys immunoassay sFlt-1/PlGF ratio, DELFIA Xpress PlGF 1-2-3 test, and BRAHMS sFlt-1 Kryptor/BRAHMS PlGF plus Kryptor PE ratio) | **DG23** |
| ImmunoCAP ISAC 112 and Microtest for multiplex allergen testing | **DG24** |
| High-throughput non-invasive prenatal testing for fetal RHD genotype | **DG25** |
| Integrated multiplex PCR tests for identifying gastrointestinal pathogens in people with suspected gastroenteritis (xTAG Gastrointestinal Pathogen Panel, FilmArray GI Panel and Faecal Pathogens B assay) | **DG26** |
| Molecular testing strategies for Lynch syndrome in people with colorectal cancer | **DG27** |
| Virtual chromoendoscopy to assess colorectal polyps during colonoscopy | **DG28** |
| Quantitative faecal immunochemical tests to guide referral for colorectal cancer in primary care | **DG30** |

**Table S2**. List of unique labels identified

| Comparative performance |
| --- |
| Comparative (diagnostic) accuracy studies |
| Consecutive recruitment |
| Cohort studies |
| Controlled clinical trials |
| Case control study/design |
| Cross-sectional study |
| Cluster randomised controlled trial |
| Diagnostic accuracy studies |
| Diagnostic cohort studies |
| Diagnostic studies with a control group |
| Discordant case analysis |
| Derivation study |
| Direct comparisons |
| End-to-end studies |
| Head to head comparisons |
| Large multi-centre RCT |
| Large prospective cohort studies collecting diagnostic accuracy data |
| Long-term studies (following patients for several years) |
| Large, multicentre prospective (UK) study |
| Multi-centre tracker study |
| Multi-centre |
| Multi-centre community-based study |
| Mixed design (of within-study comparisons) |
| Observational studies |
| Observational cohort studies |
| Pilot studies |
| Prospective study |
| Prospective cohort studies |
| Prospective, consecutive cohort study |
| Prospective, two cohorts (feasibility, validation) |
| Prospective, international multicentre |
| Primary care study |
| Population (based) studies |
| Prospective cohort randomized to either the index test or comparator |
| Prospective cohort receiving the comparator and at least one index test with follow up |
| Randomised head-to-head comparisons |
| Retrospective cohort studies |
| Retrospective studies |
| Retrospective analysis |
| Retrospective (analysis of prospective data base) |
| Randomised controlled/ non-randomised controlled trials (RCTs) |
| Single centre |
| Symptomatic study |
| Single-gate/two-gate diagnostic studies |
| Single-gate studies recruiting population-based samples |
| Single-gate studies recruiting populations at high-risk |
| Reference standard positive |
| Test-treat trials |
| Two centre |
| Two study cohorts |
| Validation study |
| Within-study comparisons |

**Table S3**. Identified key design domains and features

| **Identified design domains** | **Identified design features** | **Used terms and labels from which the features were derived:** |
| --- | --- | --- |
| Eligibility | Number of included groups | Single (incl. population based) or multiple groups (incl. single/two-gate) |
|  | Key inclusion criteria | Symptomatic, and high-risk groups/participants |
| Recruitment | Centres | From single or multiple centres |
|  | Location/care | Level of care (primary, secondary or tertiary) |
|  | Sampling of participants | Consecutive (random or convenience), retrospective or prospective recruitment |
|  | Data collection | If data is prospective or retrospective collected and/or planned to conduct/time of testing |
|  | Sampling of participants | Participants are sampled consecutively |
| Patient and test flow | Number of evaluated index test, incl. type of comparison | Comparative (incl. direct) performance |
|  | Flow of participants through tests | Receiving the comparator and at least one index test with follow up,  cohort, case-control or cross-sectional design, reference standard positive, (diagnostic) cohort, case-control |
|  | Test group allocation (randomisation) | Randomisation to either index test or comparator |
|  | The sequential order of tests performed | (diagnostic) cross-sectional design, cohort, case-control |
| Analysis | Analysis | Retrospective (post-hoc) analysis of prospective data |

The table shows all design domains and features (left column) that were identified from the DTA-specific labels (right column).

**Table S4**. List of terms to avoid and preferred terminology

| **Identified design features** | **Terms to avoid** | **Alternative, preferred terminology:** | **Clarification** |
| --- | --- | --- | --- |
| Number of included groups | Cohort study Case-control study | Single group  Two groups/gates  Multiple group/gates | Single group: included a single group with a single set of eligibility criteria  Two groups: included two with two, different sets of eligibility criteria (11) |
| Sampling of participant | Cohort study | Consecutive series Random subset Convenience series |  |
| Data collection | Prospective Retrospective | Protocol-driven data collection  Routine data collection | Protocol-specified: data collection was pre-specified in a protocol  Routine data collection: data were collected from (electronic) health records |
| Number of evaluated index tests | “Comparator” when referring to  the reference standard | Single index test  Multiple index tests  Comparative accuracy study | Comparative accuracy should only be used when two or more index tests are evaluated against the same reference standard |

The table shows the design features (column one) for which we found confusing terms (column two).

**Table S5.** Examples of when labels were used to differentiate studies

| **Labels and Context** | **Use** | **DG** |
| --- | --- | --- |
| “The majority of **studies which have adjusted for TAB**, have taken a conservative approach by excluding affected samples, **which we consider to be a reasonable practice**.”  “Use of **discordant case analysis** was inconsistent and results sometimes attributed to TAB, which may or may not be appropriate, although generally, a conservative approach of sample exclusion was used” | Labels are used to distinguish between studies that did adjustment for TAB and those that did not for the analysis (with the studies adjusting seemingly to be more reasonable than without). | GD8 |
| “**There is some concern about the generalizability** of the results of the diagnostic test accuracy systematic review, **given** **most of the studies were in high-risk or age-limited populations, rather than the unselected colorectal cancer population** which is specified in the decision problem” | Labels are used to distinguish between studies that had included high-risk populations and those that did not for the analysis (specificity was not taken from high-risk populations). | DG27 |
| “Comparisons (…) can be made using either data from studies carried out in the same study population (within-study or direct comparisons) or from data from studies where intervention and comparator tests are carried out in different populations (between-study or indirect comparisons). (…), **the preferred data for this review are derived from within-study comparisons of intervention and comparator test pathways**.” | Labels are used to distinguish between studies that had within-study comparisons and between-study comparisons for the analysis. | DG17 |
| “The inclusion criteria were expanded **to allow studies which reported direct comparisons of diagnostic accuracy**”  “One change was made to the published protocol, expanding the inclusion criteria to allow inclusion of studies which reported direct comparisons of diagnostic accuracy (…) | Labels are used to broaden inclusion criteria to other study designs. | DG24 |
| “**No randomised** or **non-randomised controlled trials** were identified. (…) **observational study** types were considered eligible for inclusion: **Cross-sectional test accuracy studies** (…). **Observational studies** (…). | Labels are used to differentiate between studies, for defining the eligible designs. | DG3 |
| “**The ideal study design** would recruit patients with symptoms of asthma, **have a cohort design** or **randomise them** (…). In the absence of such studies, **diagnostic cohort studies represent the next best level of evidence**. | Labels are used to differentiate between studies, for defining the eligible designs. | DG12 |
| “**Single-gate diagnostic studies** **with random or consecutively recruited participants were** **considered the optimal design**. (…) **Two-gate diagnostic studies** were also included.” | Labels are used to differentiate between studies, for defining the eligible designs. | DG27 |
